# Society Meetings

**Published:** 1904-09

**Authors:** 


### SOCIETY MEETINGS.

---

The American Neurological Association will hold its next annual meeting at the Planters' Hotel, Saint Louis, Thursday, Friday and Saturday, September 15, 16 and 17, 1904, under the presidency of Dr. J. J. Putnam, of Boston. Daily sessions will be held from 9 a. m. to 1 p. m. The secretary is Dr. Frank R. Fry, of Saint Louis. A general invitation to attend this meeting is extended to the medical profession.

---

The American Association of Obstetricians and Gynecologists will hold its seventeenth annual meeting at The Hotel Monticello, Saint Louis, Tuesday, Wednesday, Thursday and Friday, September 13, 14, 15, and 16, 1904, under the presidency of Dr. Walter B. Dorsett, of Saint Louis. Daily sessions will be held from 9 to 1.30; no afternoon or evening sessions will convene during this meeting, the purpose being to afford the members ample opportunity to visit the World's Fair. A cordial invitation is extended to the medical profession, resident and visiting in Saint Louis and vicinity, to attend the several scientific sessions of the association.

---

THE Fourth Pan-American Medical Congress, which was appointed to be held at Panama during the later part of December of the present year, has been postponed until the first week in January, 1905, the dates being from the fourth to the seventh of that month.

This was done at the request of many physicians who proposed to attend it, as they desired to be at home with their families during the Christmas holidays.

The delegates from this side of the continent will therefore leave on Tuesday, December 27, if they go down from New York by the regular Pacific Mail Lines, or at other dates if they go by way of New Orleans or Jamaica. The dates of sailing from the Pacific Coast have not yet been ascertained. The Congress will be held from the fourth to the seventh of January.

The officers of the Congress, appointed by President Amador, of the Republic of Panama, are: Dr. Julio Icaza, Dr. Ciro Uriola, Dr. J. Calve and Dr. Carlos Cooks, Panamanians; Dr. Gorgas, chief of the Panama Canal Sanitary Commission; Drs. Carter and Ross, Americans; Dr. Manuel Corales, Cuban; Dr. M. Stern, English, and Dr. Oduber, Dutch.

This Congress bids fair to be the most successful Pan-American Medical Congress that has ever been held, on account of the central situation of Panama and its easy approach from both sides of North America, Mexico, and the Central American Republics, as well as from the countries on the north and west sides of South America.

There will be but four sections at this Congress—surgery, medicine, hygiene and the specialties.

Further particulars may be obtained by addressing Dr. Ramon Guiteras, secretary of the International executive committee, 75 West 55th street, New York.

---

THE American Medical Editors' Association held its annual meeting at Atlantic City, June 6 and 7, 1904, under the presidency of Dr. C. E. de M. Sajous, Philadelphia. A number of new members were enrolled and several papers of interest were read. Resolutions were passed commending the action of Mr. Edward Bok, editor of the *Ladies Home Journal,* in the stand he has taken relating to the advertising and use of patented nostrums.

Memorial resolutions were adopted upon the death of Dr. Isaac N. Love the former president of the association and late editor of the *Medical Mirror*. The following-named officers for the coming year were elected: president, Harold N. Moyer, Chicago; first vice-president, C. Evelyn Pilcher, Carlisle, Pa.; second vice-president, O. F. Ball, Saint Louis; secretary and treasurer, J. MacDonald, Jr., New York; executive committee, C. E. de M. Sajous, chairman, John Punton, W. A. Young, W. C. Abbott, H. M. Simmons, C. F. Taylor and Charles Wood Fassett.

---

THE International Congress of Arts and Sciences will convene on the grounds of the Louisiana Purchase Expositions, Saint Louis, September 19-25, 1904, under the presidency of Dr. Simon Newcomb. The organisation of the congress is under the supervision of the Director of Congresses, Professor Howard J. Rogers, first assistant commissioner of education of the state of New York. There are twenty-four departments dealing with various topics relating to art and science.

After the opening of the congress on Monday afternoon, September 19, will follow, on Tuesday forenoon, addresses on main divisions of science and its applications, the general theme being the unification of each of the fields treated. These will be followed by two addresses on each of the twenty-four great departments of knowledge. The theme of one address in each case will be the Fundamental Conceptions and Methods, while the other will set forth the progress during the last century. The preceding addresses will be delivered by Americans, making the work of the first two days the contribution of American scholars.

On the third day, with the opening of the sections, the international work will begin. About 128 sectional meetings will be held on the four remaining days of the congress, at each of which two papers will be read, the theme of one being suggested by the relations of the special branch treated to other branches; the other by its present problems. Three hours will be devoted to each sectional meeting, thus enabling each hearer to attend eight such meetings, if he so desires. The program is so arranged that related subjects will be treated, as far as possible, at different times. The length of the principal addresses being limited to forty-five minutes each, there will remain at least one hour for five or six brief communications in each section. The addresses in each department will be collected and published in a special volume.

Department No. 17 deals with medicine, under the following general scheme of sections, officers and speakers:

Chairman, Dr. William Osler, Johns Hopkins University; speakers, Dr. William T. Councilman, Harvard University; Dr. Frank Billings, Rush Medical College.

*Section a. Public Health*—Chairman, Dr. Walter Wyman, Surgeon-General of the U. S. Marine Hospital Service; speakers, Prof. William T. Sedgwick, Massachusetts Institute of Technology; Dr. Ernst J. Lederle, former commissioner of health, New York City.

*Section b. Preventive Medicine*—Chairman, Dr. Joseph M. Mathews, president of the State Board of Health, Louisville, Kentucky; speakers, Prof. Ronald Ross, F. R. S., School of Tropical Medicine, University College, Liverpool; Prof. Angelo Celli, University of Rome.

*Section c. Pathology*—Chairman, Prof. Simon Flexner, Director of the Rockefeller Institute; speakers, Prof. Ludwig Hektoen, University of Chicago; Prof. Johannes Orth, University of Berlin.

*Section d. Therapeutics and Pharmacology.*—Chairman, Dr. Hobart A. Hare, Jefferson Medical College; speakers, Sir Lauder Brunton, F. R. S., London; Prof. Oscar Liebreich, University of Berlin.

*Section e. Internal Medicine.*—Chairman, Prof. Frederick C. Shattuck, Harvard University; speakers, Prof. T. Clifford Allbutt, F. R. S., University of Cambridge; Prof. William S. Thayer, Johns Hopkins University.

*Section f. Neurology.*—Chairman, Prof. Lewellys F. Barker, University of Chicago; speakers, Prof. Shibasaburo Kitasato, University of Tokio; Prof. James J. Putnam, Harvard University.

*Section g. Psychiatry.*—Chairman, Dr. Edward Cowles, Boston; speaker, Dr. Charles L. Dana, Cornell University, New York.

*Section h. Surgery.*—Chairman, Prof. Carl Beck, Post-Graduate Medical School, New York; speaker, Dr. Frederick S. Dennis, Cornell Medical College, New York City.

*Section i. Gynecology.*—Chairman, Prof. Howard A. Kelly, Johns Hopkins University; speakers, Dr. L. Gustave Richelot, Member of the Academy of Medicine, Paris; Prof. John C. Webster, Rush Medical College, Chicago.

*Section j. Ophthalmology.*—Chairman, Dr. George C. Harlan, Philadelphia; speakers, Dr. Edward Jackson, Denver; Dr. George M. Gould, Philadelphia.

*Section k. Otology and Laryngology.*—Chairman, Prof. Wm. C. Glasgow, Washington University, Saint Louis; speakers, Sir

Felix Semon, C. V. O., Physician Extraordinary to His Majesty, the King, London; Dr. J. Solis-Cohen, Jefferson Medical College.

*Section I. Pediatrics.*—Chairman, Prof. Thomas M. Rotch, Harvard University; speakers, Prof. Theodore Escherich, University of Vienna; Prof. Abraham Jacobi, Columbia University.

